# Fungal root symbionts of high-altitude vascular plants in the Himalayas

**DOI:** 10.1038/s41598-017-06938-x

**Published:** 2017-07-26

**Authors:** Milan Kotilínek, Inga Hiiesalu, Jiří Košnar, Marie Šmilauerová, Petr Šmilauer, Jan Altman, Miroslav Dvorský, Martin Kopecký, Jiří Doležal

**Affiliations:** 1 Faculty of Science, University of South Bohemia, Branišovská 1760, 370 05 České Budějovice, Czech Republic; 2 Institute of Botany, The Czech Academy of Sciences, Zámek 1, 252 43 Průhonice, Czech Republic; 3 Department of Forest Ecology, Faculty of Forestry and Wood Sciences, Czech University of Life Sciences Prague, Kamýcká 129, Prague 6, Suchdol, CZ-165 21 Czech Republic; 4 Institute of Low Temperature Science, Hokkaido University, Kita-19, Nishi-8, Kita-ku, Sapporo 060-0819 Japan

## Abstract

Arbuscular mycorrhizal fungi (AMF) and dark septate endophytes (DSE) form symbiotic relationships with plants influencing their productivity, diversity and ecosystem functions. Only a few studies on these fungi, however, have been conducted in extreme elevations and none over 5500 m a.s.l., although vascular plants occur up to 6150 m a.s.l. in the Himalayas. We quantified AMF and DSE in roots of 62 plant species from contrasting habitats along an elevational gradient (3400–6150 m) in the Himalayas using a combination of optical microscopy and next generation sequencing. We linked AMF and DSE communities with host plant evolutionary history, ecological preferences (elevation and habitat type) and functional traits. We detected AMF in elevations up to 5800 m, indicating it is more constrained by extreme conditions than the host plants, which ascend up to 6150 m. In contrast, DSE were found across the entire gradient up to 6150 m. AMF diversity was unimodally related to elevation and positively related to the intensity of AMF colonization. Mid-elevation steppe and alpine plants hosted more diverse AMF communities than plants from deserts and the subnival zone. Our results bring novel insights to the abiotic and biotic filters structuring AMF and DSE communities in the Himalayas.

## Introduction

Arbuscular mycorrhizal fungi (AMF, sub-phylum Glomeromycotina^1^) are a crucial part of soil microbiota across major biomes and associate with the vast majority of terrestrial plant species^2^. These fungi are obligate plant root symbionts that gain photosynthetic carbon from their host plant while providing it with improved nutrient uptake, resistance to pathogens and abiotic stress tolerance^3, 4^. As a result, the diversity of plants and AMF can be inter-related^5^, and AMF can have a profound effect on plant community composition and dynamics^4^. To better understand the role of AMF in shaping plant communities, more information is needed on their diversity and distribution along environmental gradients. Despite recent effort in addressing global patterns of AMF^6^, much less is known about AMF communities in extreme environments.

Some of the most extreme conditions occur in high mountains where plants are limited by low temperature, desiccation and a short growing season^7^. Although it seems that associations with AMF can be especially beneficial for plants under limited resources, previous research indicates that symbiosis is rare or dysfunctional in adverse conditions, mostly due to constraints of the carbon economy at lower temperatures^8, 9^. More recent studies have, however, found AMF colonization in host plants and diverse spore populations as high as 5200 m a.s.l.^10–12^ as well as in some of the coldest places in the world^8, 13^. Even if high-altitude mountain regions harbour relatively diverse AMF communities, it remains unclear exactly which host plants are colonized and which AMF taxa are able to persist in extreme environments. In addition to AMF, dark-septate endophytes (DSE) are often recorded in the roots of high-elevation plants^14, 15^, where they can partially or completely replace AMF^16^. DSE comprises a miscellaneous group of root-inhabiting fungi with notoriously obscure taxonomic and functional affinities. Although they can have a positive effect on plant growth^17–19^, the symbiotic status of DSE remains uncertain^20^.

Although AMF community patterns have been quite well studied in grasslands, forests and agricultural land^21^, little work has been conducted at high elevations or along elevation gradients. In addition, most studies have been concentrated in the Alps while very few reports are from elevations above 5000 m a.s.l. from the Andes and the Himalayas. Those from Andes show a rapid decrease of AMF abundance in plant roots with increasing elevation^14, 22^ while a study from an elevation gradient of 1990 to 4648 m a.s.l. in the Himalayas found no effect of elevation on AMF diversity, a significant effect on taxonomic composition and a negative effect on the abundance of AMF^23^. The highest record of AMF in the Andes comes from an elevation of about 5200 m a.s.l.^10^, however there were no studies conducted above this elevation. Similarly, there is no work from the Himalayas from elevations above 5500 m a.s.l., although there are reports about the occurrence of vascular plants up to 6150 m a.s.l.^24–26^. The studies from the Himalayas were mainly targeted on spore populations of Glomeromycotina in the soil and showed the dominance of the *Glomus* ^11, 23, 27^ or *Acaulospora* ^28^ genera. It therefore remains unknown whether AMF and DSE occur in these extreme elevations and help vascular plants to cope with the harsh environmental conditions.

We address the knowledge gap concerning AMF distribution along elevation gradients and its relation to the ecological properties of host plants by exploring the composition of mycorrhizal fungal communities associated with vascular plants growing in Ladakh, an arid high-elevation region in the NW Himalayas. Due to the dry continental climate, the mountains of Ladakh are often unglaciated up to 6200–6400 m a.s.l. allowing plant species to grow in extreme elevations, even up to 6150 m a.s.l.^7^. Environmental conditions change along the elevational gradient, ranging from a dry and hot desert, over a wide steppe belt, a water- and temperature-limited alpine belt and up to a cold, sparse subnival vegetation zone. Based on previous reports^27^, we can expect more diverse AMF communities in less stressful steppe habitats with the highest vegetation cover, and a decrease in AMF diversity towards more extreme habitats - deserts, alpine and subnival zones - with lower plant abundance. On the other hand, DSE are often recorded in the roots of plants from harsh conditions as high-elevation^29^ or arctic tundra^30^. Existing data suggest that DSE are more frequent in the plant roots in these habitats than AMF^10, 30^. Although a significant gap in knowledge exists about the functions of DSE in cold-stressed habitats, the phosphorus uptake is most probably enhanced by fine endophytes^31^ and they could be surrogate mycorrhizas in these ecosystems^32^.

In addition to environmental conditions, functional traits of host plant species, in particular those related to the carbon and water economies might influence the formation of mycorrhizal symbiosis as well as AMF community composition in plant roots^33^. Laboratory experiments showed that increasing the plant’s carbon supply to the AMF increases the uptake and transfer of phosphorus from fungi to plant^34^. Likewise, phosphorus uptake and transfer are lowered when the photosynthate supply to the fungi is decreased. Additionally, some species of AMF are poor symbionts, providing little phosphorus while taking relatively high amounts of carbon, resulting in a negative growth response^35^. Therefore, applying detailed analysis of AMF composition and diversity in tandem with host plant functional traits reflecting the carbon and water economy, may offer better insights into the processes of AMF community formation under varying environmental conditions.

One of the critical features of comparative studies on mycorrhizal association with plants and their functional traits and environmental preferences is the extent of phylogenetic relatedness among plant taxa^36^. Part of the explanatory power uncovered by relating the fungal community variation to plant ecological preferences might be alternatively explained by host plant phylogenetic inertia affecting both the similarity of fungal communities among closely related host taxa and the similarity of ecological niches that such taxa occupy. Understanding the interspecific differences in fungal communities between host species therefore requires separation of the evolutionary inertia of host plants from their true adaptation to the environment. This is commonly done by comparing analyses made with and without phylogenetic corrections^37^. This approach is based on discounting all of the variation that could possibly be explained by phylogenetic relatedness of the host species, before studying the effect of other potential predictors such as the species’ ecological niches or functional traits.

This study aimed to explore the variation in AMF community diversity and composition as well as DSE occurrence in relation to factors including altitude, habitat type, and phylogenetic relatedness as well as functional traits of host plants. We hypothesized that unlike the temperate plants that rely more on AMF in stressful and nutrient poor conditions, high-elevation plants from arid Himalayan regions will host more diverse and abundant AMF communities when they are in mesic steppe or alpine environments rather than in the extreme habitats represented by either deserts at low elevations or the cold subnival zone at high elevation. Specifically, we asked: what is the uppermost limit for AMF in the arid Himalayas? Do the habitats and their specific plants harbor specific taxa of AMF? Which abiotic and biotic factors are influencing AMF community richness and composition? Are DSE associated with the highest elevations? To answer these questions, we analyzed the AMF communities in plant roots from contrasting habitats using 454 sequencing of the SSU rRNA gene, and evaluated intra-radical AMF and DSE colonization by traditional microscopic quantification. Analyzing the roots and not the soil is an advantage in this case, as it indicates functionality rather than just identifying spores in the soil, which could very well be dormant. Although next generation sequencing (NGS) has been previously used to assess the taxonomic diversity of AMF^6^, it has not been used so far in studies from high-elevation regions. Additionally, we built a phylogenetic tree for host plant species to relate the variation in AMF community diversity and composition to host plant phylogeny. Finally, we compared in detail the variation of plant traits between mycorrhizal (*Poa attenuata*) and non-mycorrhizal (*Waldheimia tridactylites*) plant species as well as an intraspecific variation of AMF and DSE in the roots of *Poa attenuata*, both along a substantial elevational gradient.

## Results

### Plant mycorrhizal status

Out of 62 plant species studied, roots of 32 species were colonized by AMF structures (hyphae, vesicules and arbuscules) and were therefore classified as mycorrhizal plants. Two plant species (*Carex sagaensis* and *Polygonum viviparum*) had a very low level of hyphal colonization (less than 15%) with no vesicles or arbuscules present, and these were classified as non-associated with AMF. DSE was detected in 40 plant species (for details see Table S1).

**Figure 1:**
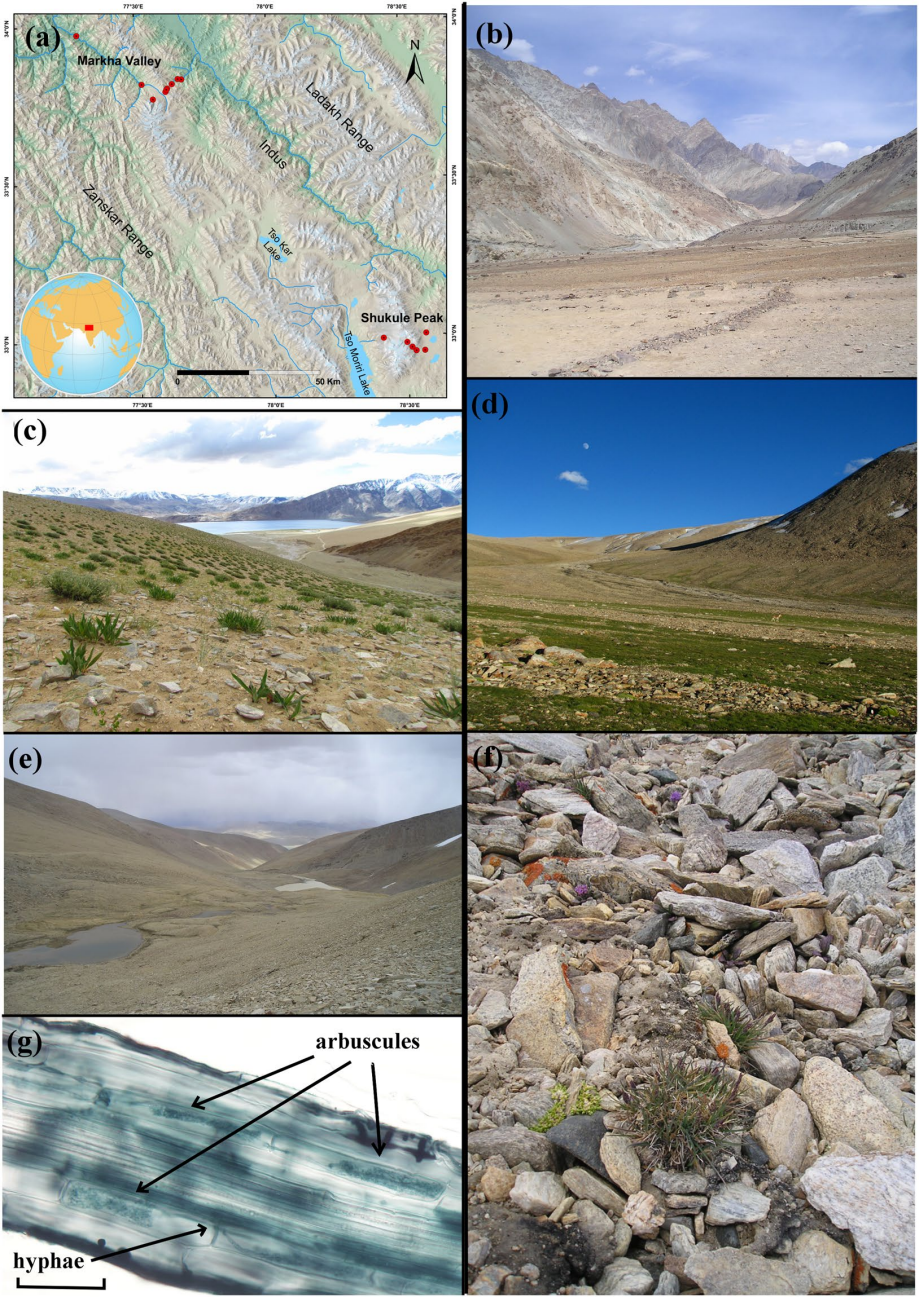
(**a**) Map of sampling sites (red point) in Ladakh, NW Himalaya. (**b**) Desert in lower part of Markha valley (3 400 m a.s.l.). (**c**) Steppe nearby the Tso Moriri Lake (4 800 m a.s.l.). (**d**) Alpine graslands nearby the Tso Moriri Lake (5 200 m a.s.l.). (**e**) Subnival vegetation on the eastern slope of the Shukule peak (5 800 m a.s.l.). (**f**) Habitat of *Poa attenuata* at 5800 m a.s.l. (**g**) Hyphae and arbuscules of AMF in a *Poa attenuata* root sample from 5800 m a.s.l. Map was generated in ArcGIS 10.2.1 (www.esri.com).

The highest-living plants with AMF in their roots were *Poa attenuata* individuals from 5800 m a.s.l., and *Saxifraga cernua* and *Saxifraga nanella* individuals from 5740 m a.s.l. (Fig. 1). From 5800 up to 6150 m a.s.l no AMF was detected, however several plant species’ roots (*P. attenuata, S. nanella* and *Stellaria decumbens*) were colonized by DSE fungi.

### Molecular identification of AMF community

We obtained a total of 52 264 quality-filtered pyrosequencing reads which belonged to 46 OTUs of Glomeromycotina (Table S2). Out of these, 43 OTUs matched (97% similarity) previously-known virtual taxa (VT; i.e a molecular operational taxonomic unit) from the MaarjAM database^21^ of published Glomeromycotina SSU rRNA gene sequences, and 3 OTUs were treated as novel VT (94–96% similarity to previously known VT belonging to Glomeraceae, Paraglomeraceae and Claroideoglomeraceae).

**Figure 2:**
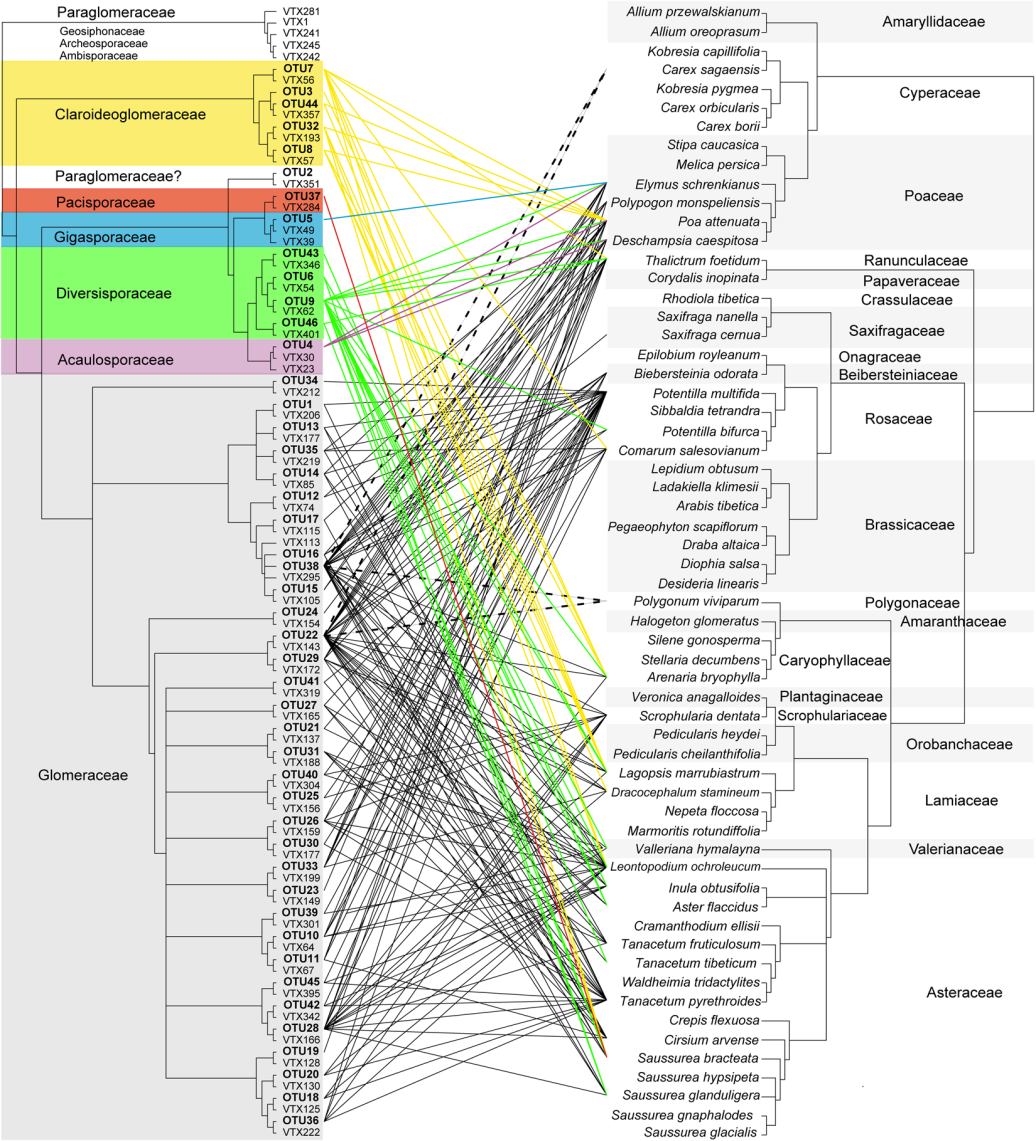
Two cladograms of Bayesian majority-rule consensus tree showing the position and relationship between recorded AMF (cladogram on left site, based on partial sequence of SSU r RNA) and host plants (cladogram on right site, based on ITS, trnT-trnL, matK + trnK, and rbcL). Connections between them are colored by fungal family: Claroideoglomeraceae (yellow), Paraglomeraceae (white), Pacisporaceae (red), Gigasporaceae (blue), Diversisporaceae (green), Acaulosporaceae (violet) and Glomeraceae (gray with black lines). Interrupted lines indicate connection between fungi and putative non-mycorrhizal plants. The AMF tree includes the detected OTUs together with the most closely related virtual taxa (VTX) from MaarjAM database. The full version of the AMF cladogram with Bayesian posterior probabilities is in Fig. S1. *Potentilla pamirica* (associated with 6 OTUs) and *Tanacetum stolickae* (associated with 2 OTUs) is omitted in the plant cladogram due to the lack of their sequences in GenBank.

**Figure 3:**
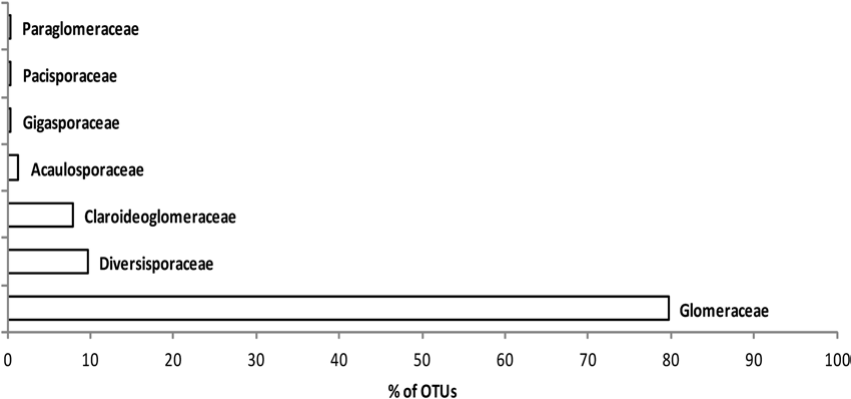
Incidence of Glomeromycotina in plant roots expressed as a percentage of OTUs (based on abundance of OTUs in samples) for particular AMF families.

From 46 OTUs, the majority (33 OTUs) belonged to Glomeraceae (*Glomus* group A). In addition, members of Claroideoglomeraceae (*Glomus* group B, 5 OTUs), Diversisporaceae (4 OTUs), Acaulosporaceae (1 OTU), Gigasporaceae (1 OTU), Pacisporaceae (1 OTU) and Paraglomeraceae (1 OTU) were found (Figs 2, 3 and S1).

### Relationships between AMF diversity, colonization and elevation

**Figure 4:**
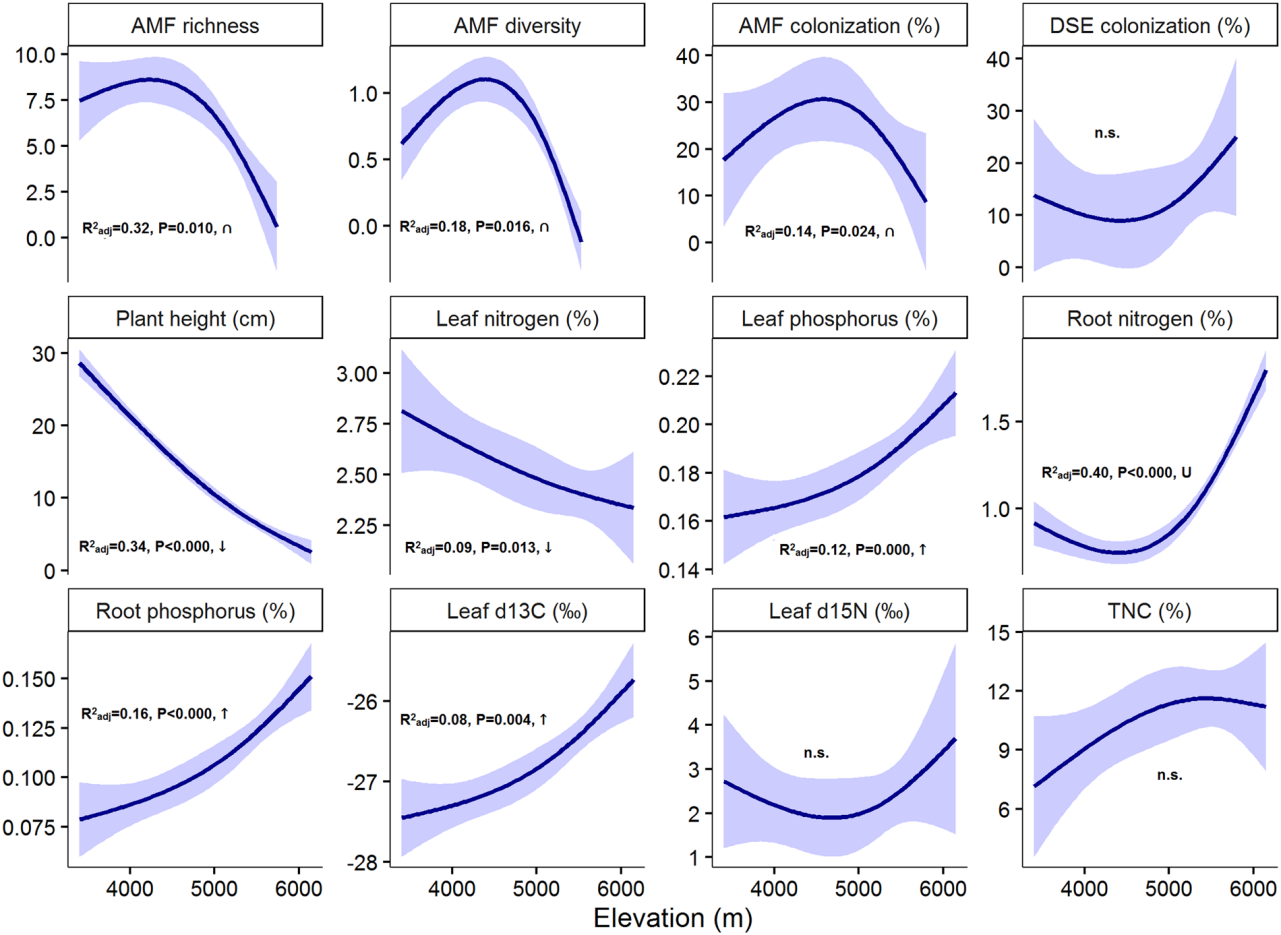
The influence of elevation on AMF attributes and plant traits analyzed using the RLM (Robust Fitting of Linear Models, thick lines), shown with 95% confidence intervals. AMF richness and diversity refers to number and Shannon diversity of OTUs. TNC - total nonstructural carbohydrates.

The highest AMF richness was found in the roots of *Potentilla multifida* (Rosaceae, 22 OTUs), *Leontopodium ochroleucum* (Asteraceae, 18 OTUs) and *Tanacetum pyrethroides* (Asteraceae, 16 OTUs). We found on average 6.5 OTUs per sample (Fig. 2 and Table S1). AMF richness did not significantly differ between plant families (Chi^2^_11_ = 11.669, P = 0.389). AMF richness and Shannon diversity, as well as AMF colonization in plant roots had a unimodal relationship with elevation, exhibiting a peak at mid-elevations (Fig. 4). AMF richness was positively related to the intensity of AMF colonization (r = 0.1421, F_1,30_ = 4.80, P < 0.05). DSE colonization intensity, however, was not significantly related to elevation (Fig. 4)

### Relationships between AMF, plant traits, environmental conditions and plant phylogeny

**Figure 5:**
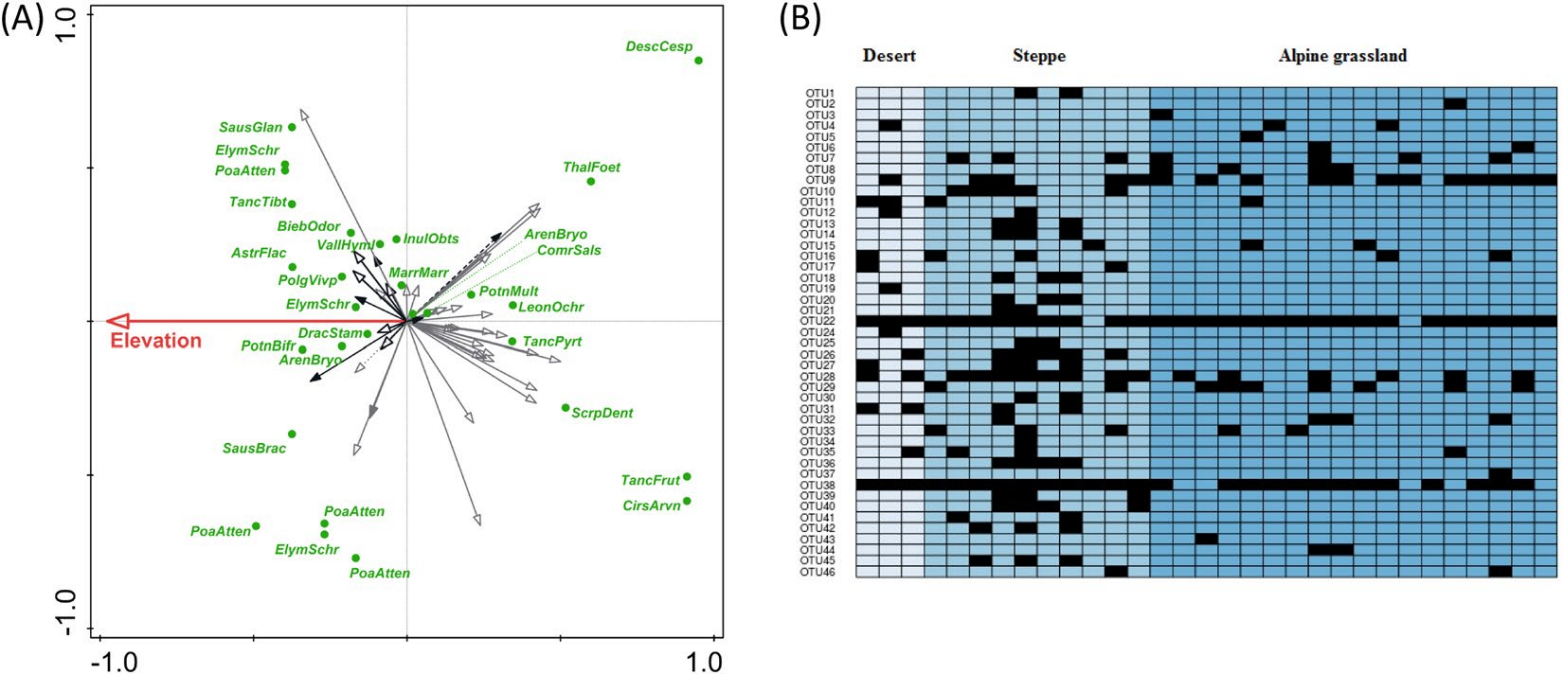
Analysis of AMF community patterns. (**A**) Ordination diagram with the first two partial RDA axes (axes explain 9.2% of the variance left after accounting for phylogenetic relations) showing the relationship between OTU composition and elevation. Small arrows point in the direction of increasing expected values of OTUs occurrence, the large arrow points in the direction of increasing expected value of elevation. The twenty best-fitted plant species are shown. AMF families are indicated by arrow type: Glomeraceae (empty grey arrows), Gigasporaceae (filled grey arrow), Acaulosporacea (filled black arrow with dashed line), Claroideoglomeraceae (empty black arrows), Pacisporaceae (empty grey arrow with dotted line), Diversisporaceae (filled black arrows). For plant taxa, the first four letters from genus and species names are used in diagram, full names are in Table S1. (**B**) Presence-absence heat map showing the occurrence of individual OTUs in different habitats.

The phylogeny-corrected RDA analyses showed a significant effect of elevation on AMF community composition in plant roots. Elevation explained 9.2% of the total variation in fungal species composition (P < 0.05, Table 1). The results of the analyses without phylogenetic corrections (hereafter ahistorical comparison) of host plant relatedness and the phylogeny-corrected analyses showed similar patterns and so only the results from the latter ones are presented in the RDA ordination diagram (Fig. 5A). The differences in the OTU composition along the first axis correspond to elevation. Members of Glomeraceae occur more frequently at lower elevation steppes and deserts. Conversely, members of Clarodieoglomeraceae and most of Diversisporaceae prevail in the higher elevations. In addition, there was a significant, unique effect of habitat type (after accounting for the effect of elevation) on AMF community composition, however, only in the analyses without phylogenetic corrections. Indicator species analyses (Fig. 5B, see also Fig. 2) identified a significant relationship between one OTU (OTU 11) and desert habitat (P < 0.01), and between 5 OTUs (OTU 10, 18, 36, 39 and 40) and steppe habitat types (P < 0.05). Two OTUs (OTU 26 and 28) were related to both of these habitats and the alpine habitat lacked indicator OTUs.

**Table 1:** Partial tests in RDA of relationships between individual traits and OTU composition (in rows), and ecological parameters (elevation and habitat with elevation as a covariate).

Factor		Evol%	Elevation			Habitat		
			AH	PC	%Ecol	AH	PC	%Ecol
Traits	All	14.7	0.016	0.010	9.5	ns	ns	14.2
	DSE	54.1	ns	ns	0.2	ns	ns	3.0
	AM	20.7	0.086	ns	2.6	0.008	0.014	9.3
	Fructan	73.2	ns	ns	0.6	ns	ns	0.4
	LCC	70.8	ns	ns	0.1	0.091	ns	0.5
	LNC	43.8	0.046	ns	0.7	ns	ns	0.8
	LPC	35.5	0.017	0.005	6.9	0.083	ns	0.1
	RNC	62.6	0.001	0.002	5.0	0.001	ns	2.0
	RPC	47.3	0.001	ns	1.9	0.006	ns	0.9
	Starch	99.1	ns	0.001	0.3	ns	ns	0.0
	FreeSug	42.8	ns	ns	0.6	ns	ns	1.4
	VF	77.5	0.001	0.001	4.2	0.001	0.075	1.7
	d13C	86.3	ns	ns	0.5	ns	ns	0.6
	d15N	35.3	ns	ns	0.7	0.006	0.093	7.6
OTU		23.1	0.038	0.034	9.2	0.039	ns	6.9

AH headed columns refer to ahistorical comparisons (i.e. without phylogenetic corrections), while PC headed columns refer to models with a correction for phylogenetic relatedness. Individual cells in AH and PC columns show Type I error probability estimates (adjusted by Bonferroni correction within test families) or ns when adjusted p ≥ 0.1. The percentage of trait variation explained by phylogenetic relatedness (Evol) between the species, and by the ecological factors (Ecol – either for Elevation or Habitat) in the phylogeny-corrected analyses, are shown. AMF – intensity of AMF colonization, DSE – intensity of DSE colonization, LCC - leaf carbon contents, LNC - leaf nitrogen contents, LPC - leaf phosphorus contents, RPC - root phosphorus contents, RNC - root nitrogen contents, d13C - leaf δ^13^C content, d15N - leaf δ^15^N content.

**Figure 6:**
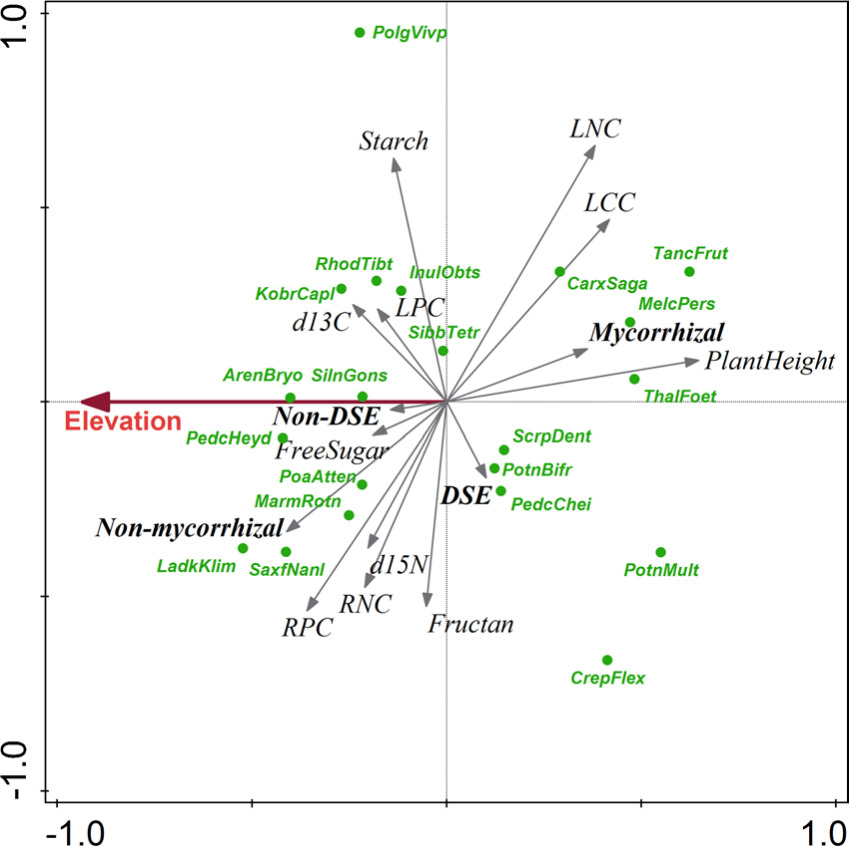
Analysis of plant functional traits. The first two partial RDA axes (after accounting for phylogenetic relations among species) showing the relationship between plant functional traits (ecophysiological parameters, AMF and DSE colonization) and elevation. Displayed axes explain 9.5% of the variance left after accounting for phylogenetic relations. Small arrows point in the direction of increasing expected values of corresponding plant traits, the large arrow points in the direction of increasing expected value of elevation. The twenty best-fitted plant species are shown. First four letters from genus and species names of host plants are used in diagram, full names are in Table S1. Full names of ecophysiological parameters are in the Table 1 legend.

In the phylogeny-corrected RDA analysis of the relationship between a set of plant traits and elevation, the first ordination axis (horizontal) separated taller mycorrhizal species (i.e. *Potentilla multifida* or *Tanacetum fruticulosum*) typical for lower elevation desert and steppe habitat from smaller, non-mycorrhizal species (e.g. *Ladakiella klimesii* or *Arenaria bryophylla*) occuring at higher elevation alpine and subnival zones (Fig. 6, Table 1). The high-elevation plants had significantly higher concentrations of phosphorus in their leaves, nitrogen and phosphorus in their roots, higher root non-structural carbohydrates and higher water use efficiencies (more negative leaf δ^13^C), compared to the plants at lower elevations (see also Fig. 4). There was a significant, unique effect of habitat type (after accounting for the effect of elevation; explained 9.3% of variability) on the distribution of mycorrhizal plants, that tended to be in deserts and steppes while non-mycorrhizal plants tended to occur in the alpine and subnival zones (Table 1).

### Poa attenuata and Waldheimia tridactylites along the elevational gradient

**Figure 7:**
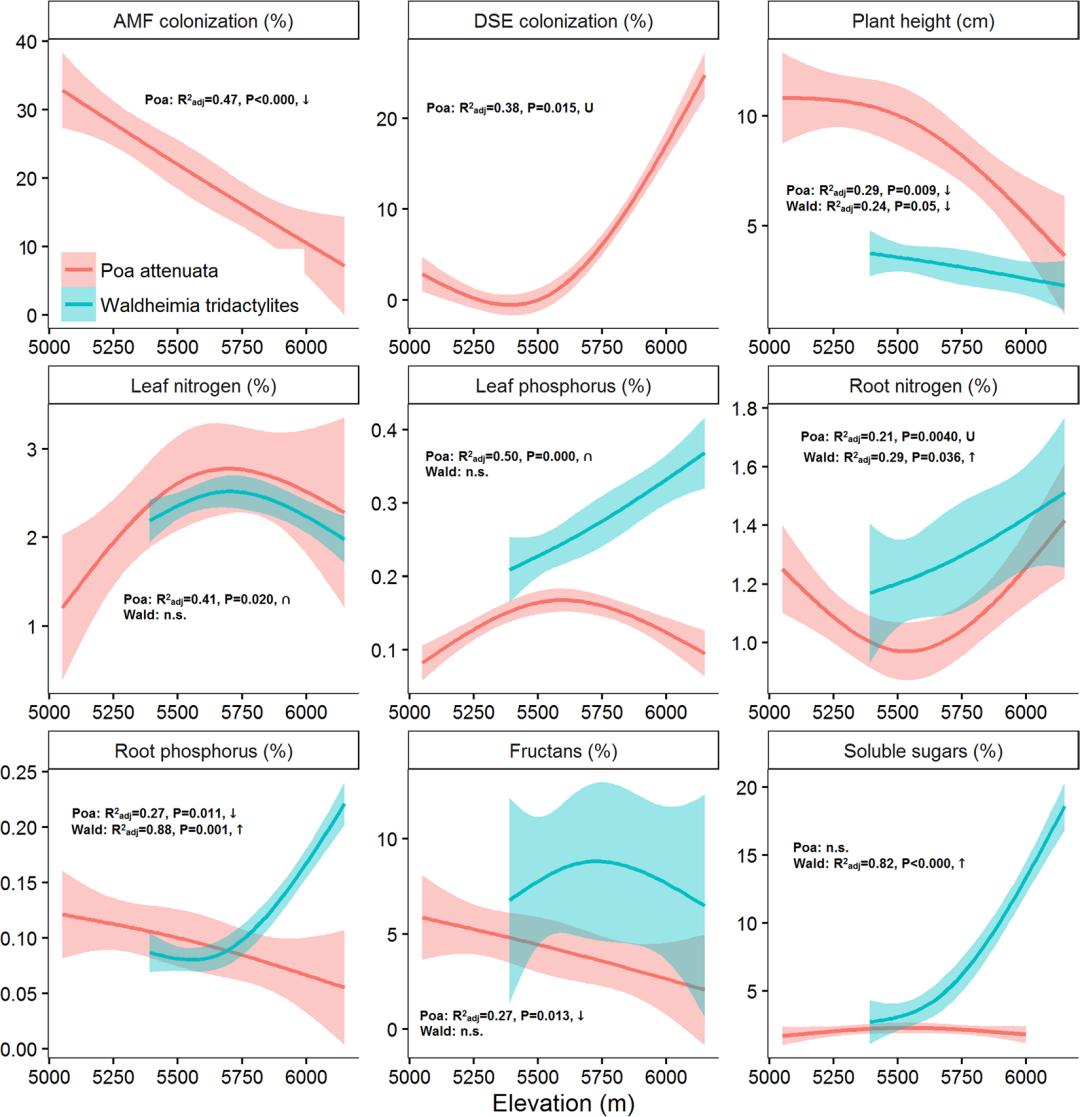
Variation in trait values for *Poa attenuata* and *Waldheimia tridactylites* on elevational gradient. The influence of elevation was analyzed using the RLM (Robust Fitting of Linear Models, thick lines) and is shown with 95% confidence intervals.

**Figure 8:**
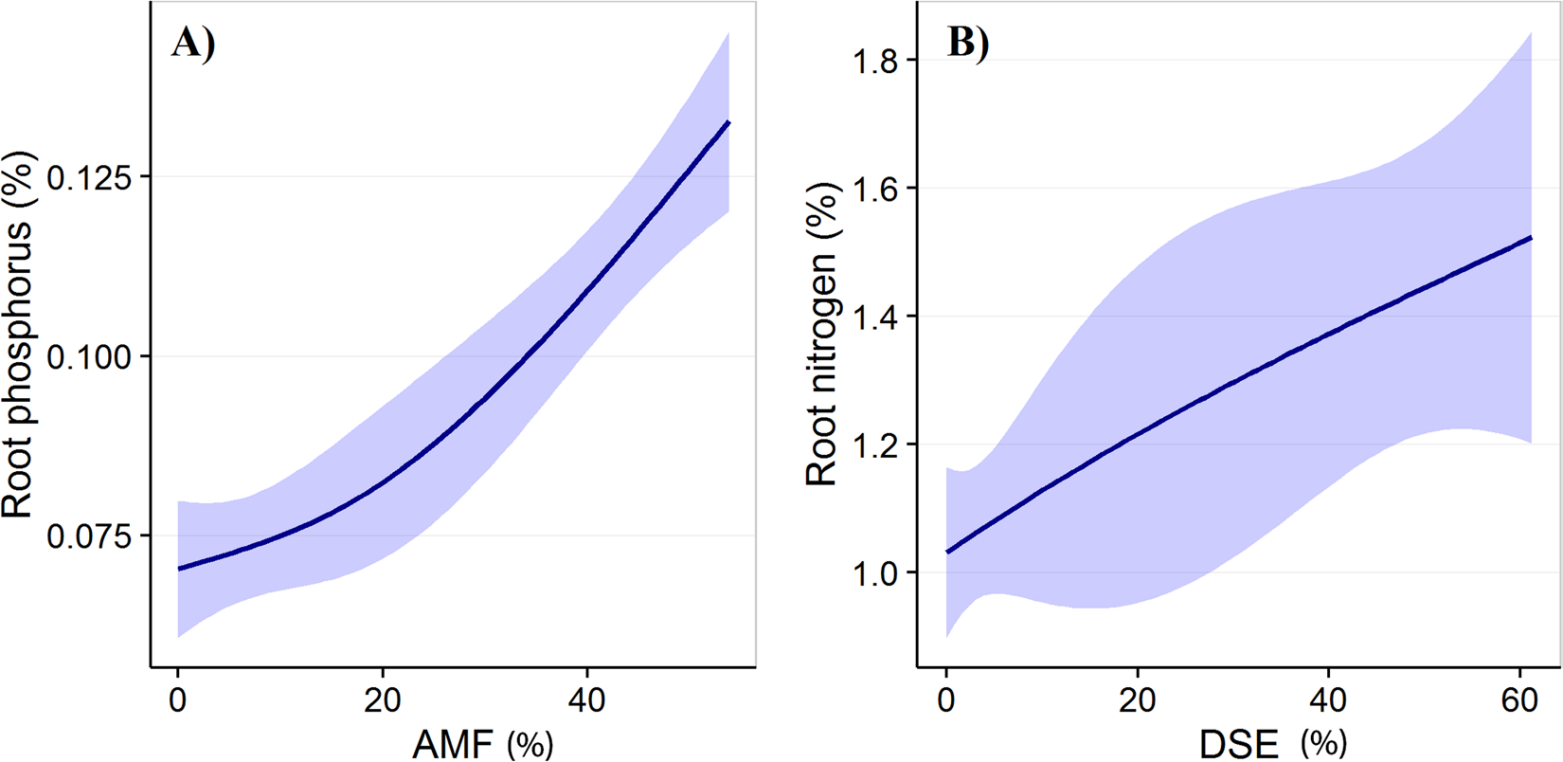
The effect of intensity of AMF and DSE colonization on nutrient content in *Poa attenuata* roots. (**A**) Correlation between root phosphorus concentration and intensity of AMF colonization (r = 0.578, n = 22, p = 0.005). (**B**) Correlation between root nitrogen concentration and intensity of DSE colonization (r = 0.572, n = 22, p = 0.005).

Although *Poa attenuata* and *Waldheimia tridactylites* both belong to potentially mycorrhizal plant families, only the roots of *P. attenuata* were intensively colonized by AMF and DSE, and no structures of AMF and a very low abundance of DSE were detected in the roots of *W. tridactylites*. While the intensity of AMF colonization in the roots of *P. attenuata* significantly decreased with elevation, the opposite pattern occurred for DSE (Fig. 7). DSE colonization was significantly negatively correlated with AMF colonization in the roots of *P. attenuata* (r = −0.745, n = 20, P < 0.001). Plant height of both species decreased with elevation. There were large differences in nutrient and carbohydrate concentrations between both species (Fig. 7). Namely, while the concentration of phosphorus, nitrogen and soluble sugars in the roots of *W. tridactylites* significantly increased with elevation, the opposite pattern was evident for *P. attenuata*. In addition, root nitrogen concentration of *P. attenuata* was positively correlated with the intensity of DSE colonization, and root phosphorus concentration with the intensity of AMF colonization (Fig. 8).

## Discussion

In order to improve the understanding of AMF distribution and diversity in extreme high-elevation environments, we analyzed AMF communities in the roots of 62 plant species from contrasting habitats along a prominent elevation gradient (3400–6150 m a.s.l.) in the dry mountains of Ladakh using a combination of optical microscopy and next generation sequencing.

As the region of Ladakh encompasses a wide range of conditions with low-elevation sites being dry and warm and high-elevation sites cold and relatively moist, plant responses bear a strong abiotic signal due to very low vegetation cover and the marginal role of interspecific interactions. These qualities of our study region allowed us to filter out major confounding factors, providing more reliable and general inferences about plant-fungal interactions along environmental gradients.

We found that high-elevation plants from the arid Himalayas host more diverse and abundant AMF communities in moderately stressful mesic steppes and alpine environments rather than in extreme habitats such as deserts at the low elevations or cold subnival zones at the highest elevations with plant life. The AMF OTU richness and intensity of colonization of plant roots declined both towards dry and cold ends of the elevational gradient. The phylogeny-corrected RDA analyses of relations between environmental conditions and intensity of colonization of mycorrhizal fungi showed significant, unique effects of habitat after accounting for elevation. Members of Glomeraceae prevailed more frequently at lower elevation steppes and deserts, while members of the Claroidieoglomeraceae and most of the Diversisporaceae were at the higher elevation alpine habitat. These results correspond with the habitat hypothesis, which postulates that both plant and AMF communities follow changes in abiotic conditions^38^.

There are several lines of evidence in our results supporting this view. For instance, AMF richness in plant roots exhibited a mid-elevation peak, similar to that previously observed for vascular plants^39^. The multivariate analyses with phylogenetic correction of host plant relatedness showed that the AMF community composition, and host plant traits covary with elevation across different habitats. Most of the results stay significant after the correction for phylogenetic relatedness of host plants, suggesting that the compositional differences of AMF are driven by host plant adaptations to temperature and moistureconstraints along the elevational gradient. Also, we detected AMF up to 5800 m a.s.l. in roots of *Poa attenuata*, but the individuals of the same species occurred without AMF up to 6150 m a.s.l., indicating that AMF are more limited by extreme conditions than their host plants^13^. This is in accordance with the positive correlation between the phosphorus content in roots of *Poa attenuata* and the intensity of mycorrhizal colonization, which corresponds to our present knowledge about AMF functioning where fungi contribute phosphorus to the plants^2^.

The rapid decrease of AMF abundance in plant roots along the elevational gradient corresponds with previous reports from high elevations^10, 14, 22, 23^. Nevertheless, all records from these studies come from elevations up to 4000 m a.s.l. and the elevation maximum of AMF occurrence is at 5500 m a.s.l.^12^. Comparing such results with studies from the Himalayas is difficult, because there are only a few reports from high elevations and most of them were focused on the diversity of AMF in the soil^11, 23, 27, 40^ that do not allow one to clarify the AMF abundance in plant roots. Analyzing the roots indicates more functional mycorrhizal associations than simply identifying spores in the soil, which could be dormant. We recorded the presence of AMF in plant roots up to 5800 m a.s.l. by using both methods - microscopy and 454 sequencing. Between 5800 and 6150 m elevation, only the two most common AMF OTUs were recorded by molecular methods in very low abundance. Simultaneously, in these samples, no arbuscules or vesicles were recorded using the optical microscope and only hyphae belonging to DSE were recorded. The positive results are probably caused by very low levels of AMF colonization. A similar case was *Polygonum viviparum*, which has been previously reported as an ectomycorrhizal (ECM)^41^, or in some cases ECM + AM plant (Wang & Qiu 2006). However, we did not detect any typical ECM nor AM structures in the roots of *P. viviparum*, but instead some hyphae. Simultaneously, we detected two OTUs from the same individuals, but of very low abundance. By combining optical microscoping with molecular methods we were able to clarify the mycorrhizal status of some plant taxa in our study area.

As expected, OTUs richness of AMF was influenced by the limitation of extreme conditions. The highest richness and abundance of AMF communities were found in mesic steppes with the most optimal conditions for plant growth compared to other habitat types (Fig. 5B). Diversity and abundance of AMF were lower in alpine and deserts habitats, and AMF presence was not recorded in the subnival zone with the most extreme conditions at 6150 m a.s.l.^24^. The absence of AMF in plant roots from the highest subnival habitats is likely caused by the conditions^7, 25^ being too extreme to allow mycorrhizal structures to establish, or by the absence of AMF in the soil. A likely reason is the short vegetation season, declining from 120 days at 5200 m a.s.l. to 40 days at 6150 m a.s.l., and a higher frequency of freeze-thaw cycles occurring^7^. This effectively reduces the time for plant assimilation, resulting in an insufficient photosynthate supply to the fungi. Moreover, phosphorus is readily available in the subnival zone, because the substrate is much wetter than in the lower elevation steppes and deserts. Elevated soil moisture, together with intensive bedrock weathering in the periglacial zone^7^ results in higher soil phosphorus concentrations, which reduces the plants’ need for AMF symbiosis. Low-temperatures in high-elevation zones restrict sink (growth) processes in plants more than the uptake of water and nutrients. Hence, the plants’ inability to use the absorbed resources for growth and tissue formation due to low-temperature restriction on enzymatic processes leads to high nutrient but also carbohydrate concentrations in alpine and subnival plants^42^. These processes could explain why our high-elevation plants have low AMF richness and abundance but high root phosphorus concentrations.

AMF absence in roots from the highest subnival habitats could also be the result of the dispersal limitation of AMF. Their large spores (up to 500 µm diameter) are only partly able to be dispersed by air, with hyphal growth allowing them to reach new substrates better than wind dispersal^2^. Due to this, AMF are often absent on initial successional stages in the glacier forefront with undeveloped soils^43, 44^, similar to the soil in subnival habitats of the studied Himalayan transect. In contrast to the glacier forefront, which predominantly connects into later successional sites occupied by AMF, the subnival zones are often fragmented by unsuitable habitats, making the colonization of new sites more difficult. On the other hand, Oehl and Körner^8^ recorded AMF in the roots and rhizospheres of isolated plants of *Saxifraga oppositifolia* on the summit of Dom in Valais (Switzerland). They suppose that wind dispersal of untypically small spores (50–90 µm) could lead to the successful AMF colonization of an unoccupied isolated site^8, 44^. Based on these controversial records, we are not able to make firm conclusion and research focusing on the dispersal of AMF and their spores in the soil is much needed.

The genus *Glomus* dominated in all studied habitats, which corroborates the other studies from the high elevation of Himalayas and elsewhere^6^. Out of 46 detected OTUs, we were unable to find a match from known VTs for three OTUs, others belonged to known virtual taxa (VT) already recorded in the MaarjAM database which includes world-wide accessions of AMF taxa^21^. In two previous studies from the Tibet plateau, 8 new AMF phylotypes (out of 21 detected) from high elevation (4500 m a.s.l.)^27^ and 8 new phylotypes (out of 38 detected) from lower elevation (3500 m a.s.l.)^45^ were reported. In comparison with the results of Gai *et al*.^11, 23^, we noted an absence of the genera *Scutellospora* and *Ambispora* and a low occurrence of genus *Acaulospora* in our samples. Although a comparison of our dataset based on molecular analyses with morphospecies detected by Gai *et al*.^11, 23^ is difficult, and the absence of *Ambispora* was most likely caused by the poor amplification of Archaeosporales by the AM1 primer^46^, the main differences in genus composition are recognizable. In addition, Liu *et al*.^27^ mentioned a similar absence of genus *Scutellospora* in their results. From the results of RDA, the Glomeraceae tend to occupy lower elevations and other families prefer higher elevations (see Fig. 5A). This is in accordance with the results of Li *et al*.^47^, who found that Glomeraceae abundance decreased with elevation while the other families increased.

We found high abundance of DSE in the roots of *P. attenuata* from alpine and subnival habitats. In addition, we found DSE reaching higher elevations than AMF. Although, the relationship between DSE root colonization intensity and elevation was not significant in our study, several studies^43, 48^ have reported an existing relationship. However, there was a significant correlation between nitrogen content in roots of *P. attenuata* and the intensity of DSE colonization, as was reported by Newsham^19^. Simultaneously, there was a correlation between nitrogen content and elevation and between DSE colonization and elevation. Therefore, our observation matches with the hypothesis that DSE might help with nutrient acquisition and could be surrogate mycorrhizas in these ecosystems^32^, although our data do not provide direct evidence about the positive effect of DSE on *P. attenuata* at extreme elevations. On the other hand, our observation is not consistent with some other studies, which show that phosphorus uptake is most probably enhanced by fine endophytes^30^. Although it could be very interesting, molecular identification of the fungal species of DSE is currently not feasible due to the absence of DSE-specific primers. Taxa of DSE could be amplified by universal fungal primers (e.g. ITS 1F^49^) together with most of the other fungal species in the plant roots, however, distinguishing between DSE and other fungi is difficult.

In summary, the highest diversity and abundance of AMF communities along the elevational gradient in the dry Himalayas were found in mesic steppes with the most suitable conditions for plant growth. Additionally, our results showed that AMF was limited by extreme conditions more than their host plants, which have their upper elevation limits at higher elevations than AMF. Species richness and colonization intensity of AMF were lower in alpine and desert habitats, and no AMF was recorded in the subnival zone above 5800 m a.s.l. with most extreme conditions. Although the mycorrhizal symbiosis is considered beneficial in stressful environments, it was absent at the highest elevations. This may be caused by the dispersal limitation of AMF in soils or by conditions too extreme to allow the establishment of the mycorrhizal structures. Conversely, DSE were found rather evenly throughout the whole elevation gradient and in all habitats, however, their benefits for plants remain unclear.

## Material and Methods

### Study area

The study was conducted in the Himalayan Mts. of Ladakh, Jammu and Kashmir State, India. The Ladakh region is arid (Leh: 115 mm/yr, 3514 m a.s.l., ca. 170 km NW of the study region, Gar: 54 mm/yr, 4232 m a.s.l., ca. 160 km SE of the study region). The studied localities ranged from 3400 m in Markha Valley to 6150 m in Tso Moriri Lake area (Fig. 1). Desert and semi-desert occupy the (relatively) lowest elevations of the Indus Valley and its major tributaries (ca. 2900–3800 m a.s.l.). The subalpine vegetation belt stretches from approximately 3800 up to 5000 m a.s.l. in east Ladakh, and is widely dominated by cold steppe vegetation. The alpine belt extends between ca. 4500 and 5200 m (occasionally to 5500 m) a.s.l., on the Tibetan border, and hosts alpine grasslands, including the characteristic moist alpine turf of *Kobresia pygmaea*. A very sparse subnival vegetation zone is developed up to 6150 m a.s.l.

The elevational gradient in our study region is tightly related to a temperature/drought gradient^39, 50^. It is therefore warmer at low-elevation deserts, with intense drought due to lower precipitation and colder and more humid at high-elevation alpine and subnival zones. The length of the growing season decreases linearly with increasing elevation, from 270 to 30 frost-free days, while mean growing season temperature decreases from 13 to 3 °C between 3400 and 6150 m^50^. Daily temperatures in the study area vary from 0 to 30 °C in summer and from −40 to −10 °C in winter. Annual precipitation is about 50 mm in the desert and semi-desert belt (falling mostly during the Indian summer monsoon), 100 mm in the alpine steppes, and 150–250 mm in the alpine and subnival zones^50, 51^. Precipitation above 5000 m a.s.l. is mostly falling as snow and its more frequent in summer than in winter^7^.

Soils have a coarse-grained structure, with a high percentage of large gravel, low water and organic matter content, high pH (7–8) and relatively high concentrations of total N and P^52^. The organic matter content and concentrations of total nitrogen, ammonia, nitrates and phosphorus increase with increasing elevation, with the highest values in the alpine zone and the lowest in the desert and semi-desert^7, 53^. The concentration of calcium and magnesium and the pH decreases with increasing altitude. The coarsest soils are found in subnival zone, where the fraction >0.5 mm represents up to 60% of the soil particles; the soil with highest proportion of fine particles is from alpine meadows.

### Plant sampling

From each site we collected one to twelve common species, represented usually by three individuals per species. In addition, *Poa attenuata* (Poaceae) and *Waldheimia tridactylites* (Asteraceae) were sampled at 150–200 m intervals on an elevation gradient from 5050 to 6150 m a.s.l. on the eastern slope of Shukule Peak above lake Chilling Tso. Fresh, non-damaged and fully developed plants at a comparable phenological stage were collected during the peak of the growing season, weighed fresh and then sun-dried *in situ*. Altogether, 219 individual plants of 62 plant species were collected. For the details see Table S1 (Supporting information).

### AMF and DSE quantification

We collected fine roots from each sampled plant and stored the samples in silica gel until processing. Approximately one-half of each sample was used for the quantification of the intra-radical AMF and DSE, whereas the other half was stored for DNA-based AMF taxon identification. The roots for fungal quantification were stained with Chlorazol Black E (Sigma-Aldrich, USA) using a modified protocol of Vierheilig *et al*.^54^: rehydrated roots were cleaned in 10% KOH at room temperature for 16 h, neutralized in 3.5% HCl for 2 min, and stained using 0.03% w/v of Chlorazol Black E in lactoglycerol (14:1:1 lactic acid, glycerol and deionized water) in a 90 °C water bath for an hour. Root AMF and DSE colonisation was quantified under a light microscope BX-50 (Olympus, Japan) in a standard light field at ×40–200 magnification, with the morphological details of AMF structures observed at ×400 magnification. To record the intensity of root colonization, we estimated a four categorical variable: total mycorrhizal colonization (six classes), the abundance of arbuscules and vesicles (four classes for both), and DSE colonization (six classes); and used them to calculate the intensity of root colonization according to a modified formula of Trouvelot *et al*.^55^, for details see supporting information A1. DSE structures were defined as clusters of inflated, rounded and thick-walled cells (microsclerotia) and melanized and septate hyphae within the cortical cells of roots as described in Jumpponen and Trappe^56^. The abundance of arbuscules (r = 0.693, n = 84, P < 0.001) and vesicles (r = 0.517, n = 84, P < 0.001) significantly positively correlated with mycorrhizal colonization, so only the total mycorrhizal colonization was used for statistical analyses. Plants with hyphal colonization more than 15% and presence of arbuscules or vesicles were considered as mycorrhizal. All samples from plant species where mycorrhiza was observed were further used for molecular identification of AMF taxa.

### Molecular identification of AMF

To assess the taxonomic composition of AMF, the collected root samples of the same species and from the same site were pooled (42 pooled samples in total, belonging to 30 plant species). Total DNA was extracted by CTAB method as described by Doyle & Doyle^57^ and purified using the PowerClean DNA Clean-Up Kit (MO BIO Laboratories, USA). A partial sequence of the small subunit (SSU) rRNA gene was amplified using primers NS31 (universal eukaryotic^58^) and AM1 (specific to AMF^59^). PCR products were used as a template for a secondary, semi-nested PCR using the universal eukaryotic primer WANDA^60^ fused with 454 Adaptor A and multiplex identifier (published by 454 Life Sciences Corporation/Roche Life Science, USA, http://www.454.com/), and the reverse primer AM1 fused with 454 Adaptor B. Both PCRs were conducted with Plain Combi PP Master Mix (Top-Bio, Czech Republic) under a temperature profile of 94 °C for 3 min, followed by 35 cycles at 94 °C for 30 s, 62 °C for 1 min, 72 °C for 40 s, and a final extension at 72 °C for 10 min for the first PCR and 94 °C for 3 min, followed by 10 cycles at 94 °C for 30 s, 60 °C for 1 min, 72 °C for 35 s, and a final extension at 72 °C for 10 min for the secondary PCR. We successfully amplified 40 samples belonging to 28 plant species. The PCR products were gel-purified using the UltraClean GelSpin DNA Extraction Kit (MO BIO Laboratories, USA), pooled at equimolar concentration, and subjected to unidirectional sequencing on a GS Junior System using Titanium Series reagents (454 Life Sciences Corporation ⁄ Roche Life Science, USA) at the Institute of Soil Biology (Biology Centre ASCR, Č. Budějovice, Czech Republic). To achieve directionality of the sequencing, DNA template was added to the A-beads only.

Sequence reads were processed using SEED 1.2.1_64bit^61^ and Mothur v.1.36^62^. Sequences were trimmed based on a minimum Phred score of 25, averaged over 50 bp moving window and chimeric sequences were detected by the UCHIME^63^. Only reads with a minimum of 400 bp were included and these were trimmed to a maximum length of 510 bp. Operational taxonomic units (OTUs) were delimited using USEARCH^64^ and 97% similarity threshold, and singletons were removed. Putative fungal species identity ware assigned using BLASTn search against the NCBI database (http://www.ncbi.nlm.nih.gov). Fungi belonging to Glomeromycotina were classified using BLASTn search against the MaarjAM database (November 2015)^21^. One representative sequence of each Glomeromycotina OTU was deposited in GenBank (Table S2). Samples which yielded more than 10 AMF sequences were used for further statistical analyses.

### Phylogenetic analysis

Phylogenetic relationships were analyzed using Bayesian inference. The phylogenetic position of AMF symbionts was analyzed using the partial sequence of SSU rRNA of one representative sequence per OTU. The obtained sequences, their close BLAST matches from MaarjAM database, and AMF sequences from GenBank covering main phylogenetic group were used for the analysis (see Table S2 for GenBank accession numbers). In total 98 sequences were aligned using the E-INS-i algorithm in MAFFT v6 (http://mafft.cbrc.jp/alignment/server^65^) and adjusted manually, yielding an alignment of 506 positions. The GTR model with auto-correlated discrete gamma distribution^66^ was selected by evaluating the AIC values in Kakusan4 v2^67^. Two runs of 5 × 10^6^ generations with sampling every 1000th generation were conducted in MrBayes v3.2.5^68^ under the best model assumption. The analysis was considered complete when the average standard deviation of split frequencies dropped below 0.01. The first 25% of trees were discarded as burn-in with the majority-rule consensus tree being built from the remaining trees. Branches with Bayesian posterior probabilities (BPP) below 0.95 were regarded as poorly supported.

For building the plant phylogeny tree, the sequences were extracted from GenBank (www.ncbi.nlm.nih.gov/nuccore/) and a combined multigene approach was applied. The maximum data coverage was achieved for the internal transcribed spacer (ITS) of nuclear ribosomal DNA and three chloroplast loci: trnT-trnL intergenic spacer, matK + trnK region, and the gene for rubisco large subunit (rbcL). The GTR model with autocorrelated discrete gamma distribution was selected as the best substitution model and two runs of 5 × 10^6^ generations with sampling every 1000th generation were conducted. The analysis was considered complete when the average standard deviation of split frequencies dropped below 0.01. The first 25% of trees were discarded as burn-in with the majority-rule consensus tree being built from the remaining trees. The nomenclature follows the Ladakh plant list^69^.

### Plant functional traits measurements

We measured several morphological and ecophysiological traits on the same plant individuals from which the fine roots were collected. These traits were: plant height, leaf carbon, nitrogen and phosphorus concentrations, leaf δ ^13^C, root nitrogen and phosphorus concentrations, and the content and composition of nonstructural carbohydrates. After transport to the laboratory the leaves and roots were oven dried (60 °C) until constant weight, ground and analyzed. Phosphorus was determined colourimetrically after digestion in HClO_4_ using a UV - 1650PC spectrophotometer (Shimadzu, Japan). The δ13 C in the plant leaves, as well as total carbon and nitrogen in the plant leaves and roots, were measured using an elemental analyser coupled to an IRMS at the Stable Isotope Facility, UC Davis, USA (http://stableisotopefacility.ucdavis.edu/). The δ^13^C values were used as an integrated, long-term measure of the ratio between internal and ambient CO_2_ concentrations (Ci/Ca), which reflects the intrinsic water use efficiency of the plants^70^. Fructan and starch contents were determined colourimetrically, and free sugars were quantified using a high-performance anion exchange chromatography with a pulsed amperometric detection^71^.

### Statistical analyses

The relationship between the AMF community parameters (OTU composition) and host plant species habitat parameters (elevation and habitat type) was evaluated using multivariate redundancy analysis (RDA^72^) fitted for OTU community data as a response variable in a global multivariate test. The relationship between plant functional traits (expressed by ecophysiological parameters of leaves and roots, and binary values of AMF and DSE colonization) and habitat parameters (elevation and habitat type) were evaluated using RDA fitted for trait data as a response variable in a global multivariate test. Elevation or habitat with elevation as a covariate were used as explanatory variables. Type I errors were estimated using non-parametric Monte Carlo permutation tests, based on the F statistic, with 1999 random permutations. We used the method by Diniz-Filho *et al*.^73^ as modified by Desdevises *et al*.^37^ for phylogenetic corrections. There, the variation explained by the phylogenetic relatedness of host species was removed from the model, using species coordinates on selected axes of a principal coordinate analysis (PCoA, calculated for both models separately) calculated from a patristic distance matrix corresponding to the MrBayes phylogenetic tree described above. Selected principal coordinates, which were used as covariates in tests including phylogenetic correction, were also used as predictors for fungal community composition (first model), as well as for functional traits and AMF and DSE colononization (second model), to estimate the amount of variation in the trait values explained by species evolutionary history^37^. All multivariate analyses were done using Canoco 5^74^.

All other analyses were performed by R software, version 3.2.3^75^. The Kruskal-Wallis test was used to compare OTU richness between plant families. Correlation between nutrient content and intensity of DSE and AMF colonization were quantified by Pearson linear correlation. The *ape* ^76^ package was used for the creation of phylogenetic trees. Indicator species analyses was performed with the *indicspecies* ^77^ package using the *multipatt* function with 999 permutations. To assess whether the attribute (OTU richness, OTU diversity - based on Shannon diversity index computed from OUT abundance, intensity of AMF and DSE colonization, functional traits) responses to elevation gradient were linear or nonlinear, a first (straight line) and second (quadratic model) order polynomial was fitted and the trend (increase, decrease, hump, or valley shape) was recorded. Response curves of the individual parameters were fitted using RLM (Robust Fitting of Linear Models). This was conducted by iterated re-weighted least squares using R package MASS^78^. Statistical differences between tested categories (e.g. age classes, NSC compounds) were based on 95% confidence intervals calculated for each the RLM line, with non-overlapping intervals indicating statistically significant differences.

## Electronic supplementary material

Supplementary material
