# Supplementary material for: Fungal root symbionts of high-altitude vascular plants in the Himalayas
Source: Sci Rep. 2017 Jul 26;7:6562. doi: 10.1038/s41598-017-06938-x (PMC5529584; doi:10.1038/s41598-017-06938-x)
Supplement: Supplementary file 1 — Supplementary material [file 41598_2017_6938_MOESM1_ESM.doc]

**Fungal root symbionts of high-altitude vascular plants in the Himalayas**

Milan Kotilínek^1*^, Inga Hiiesalu^2^, Jiří Košnar^1^, Marie Šmilauerová^1^, Petr Šmilauer^1^, Jan Altman^2^, Miroslav Dvorský^2^, Martin Kopecký^2,3^, Jiří Doležal^1,2^

1 Faculty of Science, University of South Bohemia, Branišovská 1760, 370 05, České Budějovice, Czech Republic.

2 Institute of Botany, The Czech Academy of Sciences, Zámek 1, 252 43, Průhonice, Czech Republic

3 Department of Forest Ecology, Faculty of Forestry and Wood Sciences, Czech University of Life Sciences Prague, Kamýcká 129, Prague 6 – Suchdol, CZ-165 21, Czech Republic

**A1:Estimation of AMF colonisation**

Estimation of mycorrhizal colonization according to Trouvelot et al.^55^:

Stained root fragments (F) were placed on slide and observe under the microscope and rated according to the range of classes:

M (AMF colonization) and D (DSE colonization): 0 (0%), 1(<1%), 2(1-10%), 3(10-50%), 4(50-90), 5(>90%).

A (abundance of arbuscules) and V (abundance of vesicles): 0 (0%), 1(<20%), 2(20-80%), 3(>80%)

These classes give a rapid estimation of the level of mycorrhizal and DSE colonization of each root fragment and the abundance of arbuscules and vesicles.
Based on these values, we calculated the parameters: %M, %m, %a, %A, %v, %V, D%, d% according to modified formula of Trouvelot et al.^55^.

- - Intensity of the mycorrhizal colonisation in the root system
    M% = (95*M5+70*M4+30*M3+5*M2+M1)/( total F)
    where M5 = number of fragments rated 5; M4 = number of fragments 4 etc.
  - Intensity of the mycorrhizal colonisation in the AMF-colonised fraction of roots
    m% = M*(total F)/(mycorrhizal F)
  - Arbuscule abundance in mycorrhizal parts of root fragments
    a% = (100*mA3+50*mA2+10*mA1)/100
    where mA3, mA2, mA1 are the % of m, rated A3, A2, A1, respectively, with mA3=((95*M5A3+70*M4A3+30*M3A3+5*M2A3+M1A3)/ myco F)*100/m and the same for A2 and A1.
  - Arbuscule abundance in the root system
    A% = a*(M/100)
  - Vesicle abundance in mycorrhizal parts of root fragments
    v% = (100mV3+50mV2+10mV1)/100
    where mV3, mV2, mV1 are the % of m, rated V3, V2, V1, respectively, with mV3=((95*M5V3+70*M4V3+30*M3V3+5*M2v3+M1V3)/ myco F)*100/m and the same for V2 and V1.
  - Vesicle abundance in the root system
    V% = v*(M/100)
  - Intensity of the DSE colonisation in the root system
    D% = (95*D5+70*D4+30*D3+5*D2+D1)/( total F)
    where D5 = number of fragments rated 5; D4 = number of fragments 4 etc.
  - Intensity of the DSE colonisation in the the DSE-colonised fractions of roots
    d% = D*(total F)/(DSE F)

**Table S1** Table with means of trait values for sampled species and elevations.

Legend: Altit. - Altitude, N.P. - Number of collected plants, AM - confirmed AMF colonization, DSE - confirmed DSE colonization, AM% - average intensity of AMF roots colonization, DSE% - average intensity of DSE roots colonization, OTU - number of recorded AMF OTUs. ? - the species with uncertain mycorrhizal status, * - samples used for molecular methods, however no PCR were obtained

| Plant species | Altit. | Habitat | Family | Site | N.P. | AM | DSE | AM% | DSE% | OTU |
| --- | --- | --- | --- | --- | --- | --- | --- | --- | --- | --- |
| *Allium oreoprasum* | 4240 | steppe | Liliaceae | Markha Valley | 3 | 0 | 1 | 0,00 | 33,33 |  |
| *Allium przewalskianum* | 4000 | steppe | Liliaceae | Konmaru La | 3 | 0 | 1 | 0,00 | 11,67 |  |
| *Arabis tibetica* | 5000 | steppe | Brassicaceae | Konmaru La | 2 | 0 | 0 | 0,00 | 0,00 |  |
| *Arenaria bryophylla* | 5050 | alpine | Caryophyllaceae | Chilling Tso | 3 | 1 | 1 | 1,01 | 12,22 | 2 |
| *Arenaria bryophylla* | 5200 | alpine | Caryophyllaceae | Chilling Tso | 3 | 0 | 1 | 0,00 | 78,06 |  |
| *Arenaria bryophylla* | 5390 | alpine | Caryophyllaceae | Chilling Tso | 3 | 1 | 1 | 14,14 | 64,58 | 6 |
| *Arenaria bryophylla* | 5650 | subnival | Caryophyllaceae | Chilling Tso | 3 | 0 | 1 | 0,00 | 11,90 |  |
| *Aster flaccidus* | 5300 | alpine | Asteraceae | Hamaling | 3 | 1 | 0 | 14,38 | 0,00 | 4 |
| *Biebersteinia odora* | 5000 | steppe | Biebersteiniaceae | Konmaru La | 3 | 1 | 0 | 30,28 | 0,00 | 6 |
| *Carex borii* | 5300 | alpine | Cyperaceae | Hamaling | 2 | 0 | 1 | 0,00 | 32,50 |  |
| *Carex orbicularis* | 5300 | alpine | Cyperaceae | Hamaling | 2 | 0 | 1 | 0,00 | 27,50 |  |
| *Carex sagaensis* | 4240 | steppe | Cyperaceae | Markha Valley | 2 | ? | 1 | 9,17 | 37,28 | 2 |
| *Carex sagaensis* | 5730 | subnival | Cyperaceae | Tso-Moriri | 2 | 0 | 1 | 0,00 | 22,50 |  |
| *Cirsium arvense* | 3400 | desert | Asteraceae | Markha Valley | 2 | 1 | 1 | 15,38 | 20,00 | 8 |
| *Comarum salesovianum* | 4450 | steppe | Rosaceae | Konmaru La | 3 | 1 | 1 | 9,35 | 0,33 | 7 |
| *Corydalis inopinata* | 5720 | subnival | Fumariaceae | Tso-Moriri | 3 | 0 | 0 | 0,00 | 0,00 |  |
| *Cremanthodium ellisii* | 5000 | steppe | Asteraceae | Konmaru La | 3 | 0 | 1 | 0,00 | 14,29 |  |
| *Crepis flexuosa* | 3800 | desert | Asteraceae | Markha Valley | 3 | 0 | 1 | 0,00 | 8,33 |  |
| *Deschampsia caespitosa* | 3400 | desert | Poaceae | Markha Valley | 3 | 1 | 1 | 11,46 | 14,81 | 8 |
| *Desideria linearis* | 5300 | alpine | Brassicaceae | Hamaling | 2 | 0 | 0 | 0,00 | 0,00 |  |
| *Dilophia salsa* | 5300 | alpine | Brassicaceae | Hamaling | 2 | 0 | ? | 0,00 | 7,50 |  |
| *Draba altaica* | 6150 | subnival | Brasicaceae | Shukuli | 1 | 0 | 0 | 0,00 | 0,00 |  |
| *Dracocephalum stamineum* | 5200 | alpine | Lamiaceae | Konmaru La | 3 | 1 | 1 | 2,11 | 24,32 | 6 |
| *Elymus schrenkianus* | 5050 | alpine | Poaceae | Chilling Tso | 3 | 1 | 1 | 39,10 | 69,40 | 3 |
| *Elymus schrenkianus* | 5200 | alpine | Poaceae | Chilling Tso | 3 | 1 | 1 | 14,10 | 27,28 | 7 |
| *Elymus schrenkianus* | 5390 | alpine | Poaceae | Chilling Tso | 3 | 1 | 1 | 6,37 | 72,52 | 3 |
| *Epilobium royleanum* | 3800 | desert | Onagraceae | Markha Valley | 3 | 0 | 0 | 0,00 | 0,00 |  |
| *Halogeton glomeratus* | 3400 | desert | Amaranthaceae | Markha Valley | 2 | 0 | 0 | 0,00 | 0,00 |  |
| *Inula obtusifolia* | 4800 | steppe | Asteraceae | Konmaru La | 1 | 1 | 1 | 17,27 | 0,00 | 7 |
| *Kobresia capillifolia* | 5300 | alpine | Cyperaceae | Hamaling | 2 | 0 | 1 | 0,00 | 45,00 |  |
| *Kobresia pygmea* | 5700 | subnival | Cyperaceae | Tso-Moriri | 2 | 0 | 1 | 0,00 | 72,50 |  |
| *Ladakiella klimesii* | 6000 | subnival | Brassicaceae | Shukuli | 2 | 0 | 0 | 0,00 | 0,00 |  |
| *Ladakiella klimesii* | 6150 | subnival | Brassicaceae | Shukuli | 1 | 0 | 0 | 0,00 | 0,00 |  |
| *Leontopodium ochroleucum* | 4240 | steppe | Asteraceae | Markha Valley | 3 | 1 | 0 | 44,72 | 0,00 | 18 |
| *Lepidium obtusum* | 3400 | desert | Brassicaceae | Markha Valley | 3 | 0 | 0 | 0,00 | 0,00 |  |
| *Marmoritis rotundifolia* | 5000 | steppe | Lamiaceae | Konmaru La | 2 | 0 | 0 | 0,00 | 0,00 |  |
| *Marrubium marrubiastrum* | 5036 | alpine | Lamiaceae | Chilling Tso | 3 | 1 | 1 | 20,89 | 37,50 | 10 |
| *Melica persica* | 3900 | steppe | Poaceae | Konmaru La | 1 | 1 | 1 | 28,37 | 4,35 | * |
| *Nepeta floccosa* | 3400 | desert | Lamiaceae | Markha Valley | 2 | 1 | 1 | 20,22 | 32,36 | * |
| Plant species | Altit. | Habitat | Family | Site | N.P. | AM | DSE | AM% | DSE% | OTU |
| *Pedicularis heydei* | 5300 | alpine | Orobanchaceae | Hamaling | 3 | 0 | 1 | 0,00 | 50,00 |  |
| *Pedicularis cheilanthifolia* | 4240 | steppe | Orobanchaceae | Markha Valley | 3 | 0 | 1 | 0,00 | 21,67 |  |
| *Pegaeophyton scapiflorum* | 5700 | subnival | Brassicaceae | Tso-Moriri | 2 | 0 | 1 | 0,00 | 2,50 |  |
| *Poa attenuata* | 5050 | alpine | Poaceae | Chilling Tso | 3 | 1 | 1 | 51,04 | 20,61 | 6 |
| *Poa attenuata* | 5200 | alpine | Poaceae | Chilling Tso | 3 | 1 | 1 | 18,24 | 40,68 | 4 |
| *Poa attenuata* | 5390 | alpine | Poaceae | Chilling Tso | 3 | 1 | 1 | 31,25 | 27,03 | 6 |
| *Poa attenuata* | 5530 | alpine | Poaceae | Chilling Tso | 3 | 1 | 1 | 39,75 | 14,87 | 2 |
| *Poa attenuata* | 5650 | subnival | Poaceae | Chilling Tso | 3 | 1 | 1 | 13,20 | 9,13 | 2 |
| *Poa attenuata* | 5800 | subnival | Poaceae | Chilling Tso | 3 | 1 | 1 | 6,67 | 45,00 | 2 |
| *Poa attenuata* | 6000 | subnival | Poaceae | Shukuli | 3 | 0 | 1 | 0,00 | 69,30 |  |
| *Poa attenuata* | 6150 | subnival | Poaceae | Shukuli | 1 | 0 | 1 | 0,00 | 100,00 |  |
| *Polygonum viviparum* | 5300 | alpine | Polygonaceae | Hamaling | 3 | ? | 0 | 8,02 | 0,00 | 2 |
| *Polypogon monspeliensis* | 3800 | desert | Poaceae | Markha Valley | 3 | 1 | 1 | 19,86 | 12,90 | 2 |
| *Potentilla pamirica* | 5300 | alpine | Rosaceae | Hamaling | 3 | 1 | 0 | 35,38 | 0,00 | 6 |
| *Potentilla bifurca* | 5050 | alpine | Rosaceae | Chilling Tso | 3 | 1 | 0 | 66,23 | 0,00 | 3 |
| *Potentilla multifida* | 4240 | steppe | Rosaceae | Markha Valley | 3 | 1 | 0 | 25,26 | 0,00 | 22 |
| *Rhodiola tibetica* | 5300 | alpine | Crassulaceae | Hamaling | 2 | 0 | 1 | 0,00 | 22,50 |  |
| *Saussurea bracteata* | 5300 | alpine | Asteraceae | Hamaling | 3 | 1 | 0 | 60,07 | 0,00 | 8 |
| *Saussurea glacialis* | 5900 | subnival | Asteraceae | Shukuli | 3 | 0 | 0 | 0,00 | 0,00 |  |
| *Saussurea glanduligera* | 5300 | alpine | Asteraceae | Tso-Moriri | 2 | 1 | 1 | 21,67 | 47,92 | 6 |
| *Saussurea gnaphalodes* | 6150 | subnival | Asteraceae | Shukuli | 3 | 0 | 0 | 0,00 | 0,00 |  |
| *Saussurea hypsipeta* | 5730 | subnival | Asteraceae | Tso-Moriri | 3 | 0 | 1 | 0,00 | 2,33 |  |
| *Saussurea hypsipeta* | 6000 | subnival | Asteraceae | Chilling Tso | 3 | 0 | 0 | 0,00 | 0,00 |  |
| *Saussurea hypsipeta* | 6000 | subnival | Asteraceae | Shukuli | 3 | 0 | 0 | 0,00 | 0,00 |  |
| *Saxifraga cernua* | 5740 | subnival | Saxifragaceae | Tso-Moriri | 3 | 1 | 1 | 3,33 | 6,67 | 1 |
| *Saxifraga cernua* | 5800 | subnival | Saxifragaceae | Shukuli | 3 | 0 | 0 | 0,00 | 0,00 |  |
| *Saxifraga nanella* | 5740 | subnival | Saxifragaceae | Tso-Moriri | 3 | 1 | 1 | 21,75 | 6,67 | * |
| *Saxifraga nanella* | 6000 | subnival | Caryophyllaceae | Shukuli | 1 | 0 | 1 | 0,00 | 65,00 |  |
| *Scrophularia dentata* | 4240 | steppe | Scrophulariaceae | Markha Valley | 3 | 1 | 1 | 45,66 | 23,83 | 9 |
| *Sibbaldia tetrandra* | 5300 | alpine | Rosaceae | Hamaling | 2 | 1 | 0 | 23,93 | 0,00 | * |
| *Silene gonosperma* | 5300 | alpine | Caryophyllaceae | Hamaling | 3 | 0 | 1 | 0,00 | 3,33 |  |
| *Stellaria decumbens* | 6000 | subnival | Caryophyllaceae | Shukuli | 3 | 0 | 1 | 0,00 | 15,48 |  |
| *Stipa caucasica* | 3900 | steppe | Poaceae | Konmaru La | 3 | 0 | 1 | 0,00 | 100,00 |  |
| *Tanacetum fruticulosum* | 3400 | desert | Asteraceae | Markha Valley | 2 | 1 | 1 | 12,00 | 5,84 | 6 |
| *Tanacetum pyrethroides* | 4240 | steppe | Asteraceae | Markha Valley | 3 | 1 | 1 | 49,38 | 6,60 | 16 |
| *Tanacetum stolickae* | 3900 | steppe | Asteraceae | Konmaru La | 2 | 1 | 1 | 6,93 | 3,00 | 2 |
| *Tanacetum tibeticum* | 5300 | alpine | Asteraceae | Hamaling | 3 | 1 | 1 | 9,92 | 10,00 | 2 |
| *Thalictrum foetidum* | 4000 | steppe | Ranunculaceae | Konmaru La | 3 | 1 | 0 | 51,94 | 0,00 | 11 |
| *Valeriana hymalayana* | 5000 | steppe | Valerianaceae | Konmaru La | 2 | 1 | 0 | 13,84 | 0,00 | 6 |
| *Veronica anagalloides* | 3800 | desert | Scrophulariaceae | Markha Valley | 3 | 0 | 1 | 0,00 | 1,67 |  |
| *Waldheimia tridactylites* | 5390 | alpine | Asteraceae | Chilling Tso | 3 | 0 | 0 | 0,00 | 0,00 |  |
| *Waldheimia tridactylites* | 5530 | alpine | Asteraceae | Chilling Tso | 3 | 0 | 0 | 0,00 | 0,00 |  |
| *Waldheimia tridactylites* | 5650 | subnival | Asteraceae | Chilling Tso | 3 | 0 | 1 | 0,00 | 13,58 |  |
| *Waldheimia tridactylites* | 5800 | subnival | Asteraceae | Chilling Tso | 3 | 0 | 0 | 0,00 | 0,00 |  |
| *Waldheimia tridactylites* | 6150 | subnival | Asteraceae | Shukuli | 3 | 0 | 0 | 0,00 | 0,00 |  |

**Table S2** Table of recorded OTUs and their closest BLAST matches from MaajrAM database^21^. OTUs 1-3 are treated as a new VTX.

| **OTU** | **Accession number** | Closest VTX | Closest BLAST matches | | Family | Length | Score (bits) | Expected | Percent |
| --- | --- | --- | --- | --- | --- | --- | --- | --- | --- |
|  |  |  | Accession number | Description |  |  |  |  |  |
| **1** | **KY608176** | VTX206 | LN617842 | Glomus sp. | Glomeraceae | 500 | 795 | 0.0 | 96% |
| **2** | **KY608177** | VTX351 | HE576800 | Paraglomus Alguacil12b ACA1 | Paraglomeraceae ? | 510 | 854 | 0.0 | 96% |
| **3** | **KY608212** | VTX357 | HE576833 | Claroideoglomus Alguacil12b GLO G3 | Claroideoglomeraceae | 510 | 791 | 0.0 | 94% |
| **4** | **KY608213** | VTX30 | AJ306441 | Acaulospora sp. | Acaulosporaceae | 506 | 961 | 0.0 | 99% |
| **5** | **KY608214** | VTX49 | FJ009672 | Scutellospora calospora | Gigasporaceae | 502 | 957 | 0.0 | 99% |
| **6** | **KY608178** | VTX54 | LN622173 | Diversispora sp. | Diversisporaceae | 513 | 863 | 0.0 | 98% |
| **7** | **KY608179** | VTX56 | JX999434 | Claroideoglomus Shi14a Phy-11 | Claroideoglomeraceae | 510 | 977 | 0.0 | 99% |
| **8** | **KY608180** | VTX57 | FN869808 | Claroideoglomus Glo G8 | Claroideoglomeraceae | 509 | 961 | 0.0 | 99% |
| **9** | **KY608181** | VTX62 | HQ610613 | Diversispora Div-2 | Diversisporaceae | 506 | 981 | 0.0 | 99% |
| **10** | **KY608182** | VTX64 | KF467266 | Glomus Shi14b Glo-13 | Glomeraceae | 503 | 944 | 0.0 | 98% |
| **11** | **KY608183** | VTX67 | HE613469 | Glomus Alguacil14b Glo14 | Glomeraceae | 504 | 981 | 0.0 | 99% |
| **12** | **KY608215** | VTX74 | JQ654521 | Glomus Kluber12 OTU8 | Glomeraceae | 502 | 950 | 0.0 | 98% |
| **13** | **KY608184** | VTX77 | LN622478 | Glomus sp. | Glomeraceae | 505 | 987 | 0.0 | 100% |
| **14** | **KY608185** | VTX85 | LN622540 | Glomus sp. | Glomeraceae | 502 | 952 | 0.0 | 99% |
| **15** | **KY608186** | VTX105 | FN869723 | Glomus Glo G1 | Glomeraceae | 502 | 940 | 0.0 | 98% |
| **16** | **KY608221** | VTX113 | DQ263965 | Glomus sp. | Glomeraceae | 506 | 906 | 0.0 | 99% |
| **17** | **KY608187** | VTX115 | JN009217 | Glomus sp. | Glomeraceae | 503 | 954 | 0.0 | 99% |
| **18** | **KY608188** | VTX125 | LN622317 | Glomus sp. | Glomeraceae | 503 | 987 | 0.0 | 100% |
| **19** | **KY608189** | VTX128 | DQ085202 | Glomus JP3 | Glomeraceae | 504 | 934 | 0.0 | 98% |
| **20** | **KY608190** | VTX130 | LN620066 | Glomus sp. | Glomeraceae | 532 | 810 | 0.0 | 98% |
| **21** | **KY608191** | VTX137 | JX999386 | Glomus sp. | Glomeraceae | 501 | 920 | 0.0 | 98% |
| **22** | **KY608192** | VTX143 | GU238336 | Glomus Glo-A7 | Glomeraceae | 501 | 961 | 0.0 | 99% |
| **23** | **KY608216** | VTX149 | LN621150 | Glomus sp. | Glomeraceae | 501 | 920 | 0.0 | 98% |
| **OTU** | **Accession number** | VTX | Closest BLAST matches | | Family | Length | Score (bits) | Expected | Percent |
|  |  |  | Accession number | Description |  |  |  |  |  |
| **24** | **KY608193** | VTX154 | LN622557 | Glomus sp. | Glomeraceae | 510 | 879 | 0.0 | 99% |
| **25** | **KY608194** | VTX156 | JX999426 | Glomus sp. | Glomeraceae | 501 | 933 | 0.0 | 99% |
| **26** | **KY608195** | VTX159 | LN617026 | Glomus sp. | Glomeraceae | 503 | 961 | 0.0 | 99% |
| **27** | **KY608196** | VTX165 | EF154346 | Glomus sp. | Glomeraceae | 502 | 961 | 0.0 | 99% |
| **28** | **KY608197** | VTX166 | AB556923 | Glomus sp. | Glomeraceae | 502 | 969 | 0.0 | 99% |
| **29** | **KY608198** | VTX172 | LN622330 | Glomus sp. | Glomeraceae | 502 | 946 | 0.0 | 99% |
| **30** | **KY608199** | VTX177 | LN622784 | Glomus sp. | Glomeraceae | 501 | 971 | 0.0 | 99% |
| **31** | **KY608200** | VTX188 | HE775411 | Glomus Kohout14 A-11/MOTU-17 | Glomeraceae | 504 | 902 | 0.0 | 98% |
| **32** | **KY608201** | VTX193 | HG004527 | Claroideoglomus Torrecillas 13 Cl7 | Claroideoglomeraceae | 506 | 944 | 0.0 | 98% |
| **33** | **KY608202** | VTX199 | JN252455 | Glomus Early-7 | Glomeraceae | 503 | 967 | 0.0 | 99% |
| **34** | **KY608217** | VTX212 | LN617673 | Glomus sp. | Glomeraceae | 510 | 926 | 0.0 | 99% |
| **35** | **KY608203** | VTX219 | GU353412 | Glomus LER02 | Glomeraceae | 501 | 971 | 0.0 | 99% |
| **36** | **KY608204** | VTX222 | JF683576 | Glomus sp. | Glomeraceae | 502 | 969 | 0.0 | 99% |
| **37** | **KY608218** | VTX284 | HQ610612 | Pacispora Pac-1 | Pacisporaceae | 506 | 981 | 0.0 | 99% |
| **38** | **KY608205** | VTX295 | GU238357 | Glomus Glo-A2 | Glomeraceae | 503 | 973 | 0.0 | 99% |
| **39** | **KY608206** | VTX301 | HQ424237 | Glomus Liu2012a GA-5 | Glomeraceae | 502 | 969 | 0.0 | 99% |
| **40** | **KY608207** | VTX304 | LN620134 | Glomus sp. | Glomeraceae | 501 | 979 | 0.0 | 99% |
| **41** | **KY608208** | VTX319 | GU238379 | Glomus Glo-A6 | Glomeraceae | 504 | 977 | 0.0 | 99% |
| **42** | **KY608209** | VTX342 | HG004520 | Glomus Torrecillas 13 Glo G7 | Glomeraceae | 505 | 900 | 0.0 | 98% |
| **43** | **KY608219** | VTX346 | GU238392 | Entrophospora Clade-1 | Diversisporaceae | 505 | 959 | 0.0 | 99% |
| **44** | **KY608210** | VTX357 | HE576833 | Claroideoglomus Alguacil12b GLO G3 | Claroideoglomeraceae | 510 | 973 | 0.0 | 99% |
| **45** | **KY608211** | VTX395 | FM876932 | Glomus Alguacil09a Glo unk4 | Glomeraceae | 502 | 969 | 0.0 | 99% |
| **46** | **KY608220** | VTX401 | JN252443 | Diversispora Early-2 | Diversisporaceae | 505 | 924 | 0.0 | 98% |


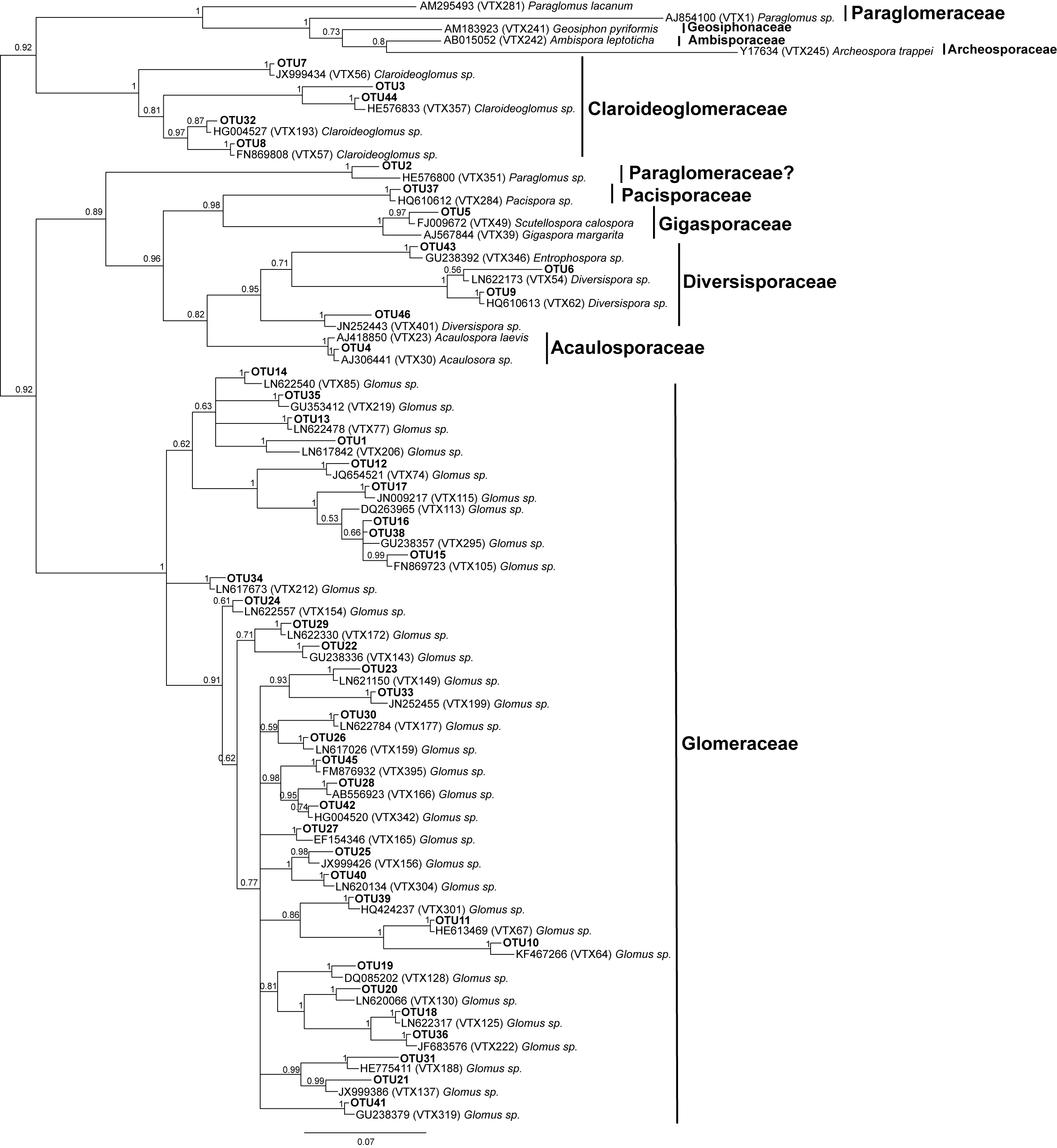
**Fig. S1** Cladogram of Bayesian majority-rule consensus tree (based on part of 18S) showing the position of recorded operational taxonomic units (OTUs) in Glomeraceae. Bayesian posterior probabilities are denoted above the branches.
